# Recovery of Ischemic Limb and Femoral Artery Endothelial Function Are Preserved in Mice with Dextran Sodium Sulfate-Induced Chronic Colitis

**DOI:** 10.3390/biology11081169

**Published:** 2022-08-04

**Authors:** Hao Wu, Qiang Zhu, Xuanyou Liu, Hong Hao, Zhe Sun, Meifang Wang, Michael A. Hill, Canxia Xu, Zhenguo Liu

**Affiliations:** 1 Center for Precision Medicine and Division of Cardiovascular Medicine, University of Missouri School of Medicine, Columbia, MO 65212, USA; 2 Department of Gastroenterology, Third Xiangya Hospital, Central South University, Changsha 410013, China; 3 Dalton Cardiovascular Research Center, University of Missouri, Columbia, MO 65212, USA

**Keywords:** inflammatory bowel disease, limb ischemia, endothelial function, ROS

## Abstract

**Simple Summary:**

The present study examines the effect of experimental inflammatory bowel disease on femoral artery endothelial function and limb ischemia recovery in female mice using a chronic colitis model induced by dextran sodium sulfate exposure. As expected, plasma levels of proinflammatory cytokines, including interleukin-6, interleukin-17, tumor necrosis factor alpha, and chemokine ligand 1, were significantly increased in the chronic colitis model. However, ROS levels in the ischemic muscle tissues were not significantly increased in mice with colitis as compared to controls. There were no significant changes in endothelium-dependent or -independent vasodilation of femoral artery between the colitis model and the control. Recovery of function and blood flow of the ischemic limb and capillary density in the ischemic muscle were preserved in the colitis model as compared with the control.

**Abstract:**

Inflammatory bowel disease (IBD) produces significant systemic inflammation and increases the risk of endothelial dysfunction and peripheral artery disease. Our recent study demonstrated that abdominal aortic endothelial cell function was impaired selectively in female mice with chronic colitis. This study aimed to test the hypothesis that experimental colitis leads to femoral artery endothelial cell dysfunction and impairs limb ischemia recovery in female mice. An experimental chronic colitis model was created in female C57BL/6 mice with dextran sodium sulfate (DSS) treatment. Unilateral hind limb ischemia was produced by femoral artery ligation. Limb blood perfusion, vascular density, tissue ROS levels, and plasma levels of proinflammatory cytokines were assessed. Femoral artery endothelium-dependent and -independent vasodilation of the contralateral limb were evaluated ex vivo using acetylcholine and nitroglycerin, respectively. As expected, the plasma levels of proinflammatory cytokines, including tumor necrosis factor alpha (TNF-α), interleukin (IL)-6, and IL-17, were significantly increased in the DSS-induced colitis model. However, ROS levels in the ischemic muscle tissues were not significantly increased in colitis model as compared to the controls. There were no significant changes in endothelium-dependent or -independent vasodilation of the femoral artery between colitis model and the control. Recovery of function and blood flow in the ischemic limb and capillary density in the ischemic gastrocnemius muscle were preserved in the colitis model as compared with the control. The data demonstrated that DSS-induced chronic colitis had no significant impact on femoral artery endothelial function or ischemic limb recovery in female mice.

## 1. Introduction

Inflammatory bowel disease (IBD), encompassing ulcerative colitis (UC) and Crohn’s disease (CD), is characterized by chronic and recurrent intestinal inflammation. IBD not only alters the function of the gastrointestinal system, but can also affect many other organ systems, which are referred to as extraintestinal manifestations (EIMs) [1,2]. Several population-based studies have previously shown that patients with IBD are at an increased risk of developing cardiovascular diseases (CVDs), including peripheral artery disease (PAD), ischemic heart disease, and cerebrovascular disease [3,4]. However, the underlying mechanisms for links between IBD and CVDs remain poorly defined.

Systemic inflammation and endothelial cell dysfunction are considered among the important factors for the development and progression of CVDs [5,6]. Several inflammatory biomarkers, including C-reactive protein, calprotectin, TNF-α, IL-6, and IL-17, are significantly increased in patients with IBD [7]. Studies have demonstrated that endothelial cell dysfunction, as reflected by decreases in flow-mediated dilatation and pulse arterial tonometry indices, is present in patients with IBD [8,9]. Consistent with this, our recent meta-analysis showed that patients with IBD were significantly associated with endothelial cell dysfunction, increased arterial stiffness, and carotid intima-media thickness [10].

Dextran sodium sulfate (DSS) induces significant damages to the intestinal epithelium. The epithelial damage compromises the barrier integrity and subsequently exposes the mucosal and submucosal immune cells to bacterial antigens, resulting in significant local and systemic inflammation [11]. Our recent study showed that abdominal aortic endothelial cell function was impaired selectively in female mice with chronic colitis [12]. The French population-based cohort study demonstrated that the risk of developing PAD was significantly increased 1.27-fold in patients with IBD compared with the general population [4]. To our knowledge, there have been no animal studies to evaluate limb ischemia recovery and femoral artery endothelial function in DSS-induced colitis. The present study was aimed to test the hypothesis that experimental colitis could lead to femoral artery endothelial cell dysfunction and impaired hind limb ischemia recovery.

## 2. Materials and Methods

### 2.1. Animals

All mice procedures were conducted in accordance with the Guide for the Care and Use of Laboratory Animals of National Institutes of Health (NIH). The protocols were approved by the Institutional Animal Care and Use Committee of the University of Missouri, MO, USA. Eight-week-old C57BL/6 female mice were ordered from Jackson Lab (Bar Harbor, ME, USA). Mice were fed standard laboratory chow and maintained under controlled conditions (22 ± 1 °C, 12 h light/12 h dark cycle) until euthanasia.

### 2.2. Chronic DSS-Induced Colitis and Critical Limb Ischemia Mouse Model

**Figure 1:**
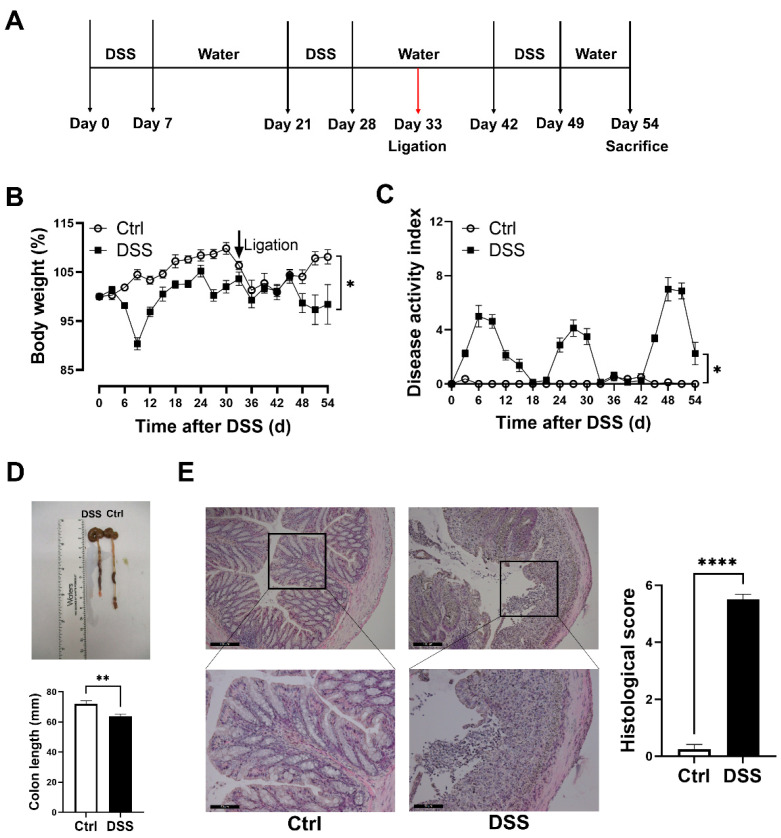
Female mice with chronic colitis displayed significant inflammation in the colon. (**A**) Experimental scheme illustrating DSS treatment and femoral ligation protocol for chronic colitis and limb ischemia models. Changes in (**B**) body weight and (**C**) DAI during DSS treatment. (**D**) Colon length and representative photographs for colon tissue in colitis and control mice. (**E**) Representative images of H&E staining of colon tissue and summary of histological score (scale bar, 100 μm and 50 μm). Results are expressed as mean ± SEM. * *p* < 0.05, ** *p* < 0.01, **** *p* < 0.0001, by unpaired 2-tailed Student’s *t*-test, *n* = 8 mice in each group. DAI, disease activity index.

To induce colitis, mice were administered with 2.5% (*wt*/*vol*) DSS solution (MP Biomedicals, Santa Ana, CA, USA) as described [11]. Mice for the chronic model were randomly assigned to either control (*n* = 8) or DSS (*n* = 8) treatment groups. Mice were treated with 3 cycles of DSS (seven days of DSS treatment with fourteen days of drinking water between each cycle) and subsequently sacrificed on day 51 as shown in Figure 1A. DSS solution was freshly made every other day. On day 33, unilateral hind limb ischemia was produced by femoral artery ligation as previously described [13]. In brief, mice were anesthetized with 2% isoflurane/O_2_, and placed in supine position over a heated pad to maintain body temperature. The left femoral artery was exposed through a 1 cm incision, and then separated from the femoral vein. The proximal femoral artery was occluded and the distal femoral artery proximal to the popliteal artery was ligated with 7–0 suture. The segment of femoral artery between the proximal and distal knots was transected, and the incision was closed using 5–0 Vicryl sutures.

### 2.3. Analysis of Colitis and Ischemic Limbs

All mice were weighed every three days and evaluated for fecal occult blood using Compliance Gold (Germaine Laboratories, San Antonio, TX, USA) and stool consistency. The colitis severity was determined using disease activity index (DAI) for each mouse as described [14]. At the day of euthanasia, colon length was measured from the anal verge to the ileocecal junction. Gastrocnemius muscle and colon tissues were collected and embedded in paraffin and cut into 5 μm-thick tissue sections. The colon tissue and gastrocnemius muscle sections were stained with hematoxylin and eosin (H&E) and examined using an automated upright microscope system (Leica DM5500B, Wetzlar, Germany). The level of inflammation in colon tissue was determined using a scoring system for inflammation-associated histological changes as described [11]. The degeneration of gastrocnemius muscle was examined in the H&E-stained sections morphologically.

### 2.4. Analyses of Plasma Cytokines

While under isoflurane anesthesia, blood was collected from each mouse into K3 EDTA micro tubes (Sarstedt, Nümbrecht, Germany). Each blood sample was centrifuged at 3000× *g* for 10 min at 4 °C to obtain the plasma. The levels of plasma cytokines were measured using a mouse cytokine/chemokine discovery assay (Eve Technologies, Calgary, AB, Canada) [15].

### 2.5. Laser Doppler Perfusion Imaging and Function Assessment of Ischemic Limb

Limb blood flow was measured noninvasively using a laser doppler perfusion imaging system (Moor Instruments, Wilmington, DE, USA) as described [16]. Briefly, the evaluation of limb perfusion was conducted by acquiring flow images of the ischemic and contralateral limb before the creation of limb ischemia (baseline), and on days 0, 3, 7, 14, and 21 post-ischemia. After removal of excessive hair from the limbs, mice were placed on a heating pad at 37 °C to minimize temperature variation. The flux ratio of the total blood perfusion (ischemic limb blood flow/control limb blood flow) was used to determine the recovery of limb circulation. Assessments of limb function and ischemia were conducted at the same time points as blood flow measurements. Limb function was evaluated using the Tarlov scale and limb ischemia was assessed using the modified ischemia scale, as described [17,18].

### 2.6. Analysis of Vascular Density and ROS Production

Gastrocnemius muscle tissues of the ischemic limb were collected and embedded in optimal cutting temperature compound (Sakura Finetek, Torrance, CA, USA), and frozen in liquid nitrogen immediately. Tissue vascular density was evaluated using CD31 immunofluorescent staining as described [19]. Briefly, the frozen cross sections (5 μm in thickness) of gastrocnemius muscle tissue were sequentially fixed with 4% paraformaldehyde for 10 min, washed with PBS (x3), and blocked with 5% bovine serum albumin in PBS for 30 min at room temperature. After washing with PBS, the sections were incubated with Alexa Fluor 594 anti-mouse CD31 immunofluorescent antibody (1:500, Biolegend, San Diego, CA, USA) at 4 °C overnight. After rinsing in PBS (x3), the sections were incubated with 4′,6-diamidino-2-phenylindole (DAPI) for 10 min at room temperature, and then washed in PBS (x3), and examined using a fluorescence microscope (Olympus CKX53, Tokyo, Japan). Vascular density was determined using Image J software (NIH, Bethesda, MD, USA) with VesselJ plugin [19]. Four independent fields were examined for each section to calculate the mean vascular density. Tissue ROS levels were measured using dihydroethidium assay, as described [20]. Briefly, the frozen sections (5 μm in thickness) of gastrocnemius muscle tissue were prepared and incubated with 5 μM dihydroethidium (DHE; Molecular Probes, Eugene, OR, USA) for 15 min. Images were captured using a fluorescence microscope (Olympus CKX53, Tokyo, Japan), and analyzed with Image J software to calculate the mean gray value.

### 2.7. Evaluation of Femoral Artery Endothelial Function Ex Vivo

The femoral artery of the contralateral limb was carefully excised and prepared in ice-cold physiological saline solution (PSS) containing (in mM) 130 NaCl, 4.7 KCl, 14.9 NaHCO_3_, 1.6 CaCl_2_, 1.18 KH_2_PO_4_, 1.17 MgSO_4_, 5.5 glucose, and 0.026 EDTA, pH 7.4, gassed with 5% CO_2_ and 95% O_2_. The femoral artery section was then carefully cut into rings (2 mm in length) under a stereo microscope. The arterial rings were mounted in a wire myograph system (620M; Danish Myo Technology, Hinnerup, Denmark). The organ chambers were filled with 5 mL PSS at 37 °C and aerated continuously with carbogen (95% O_2_ + 5% CO_2_). Based on vessel length/tension relationships, a preload tension of 3 mN/mm was applied to the rings. The vascular preparations were allowed to equilibrate for one hour with replacement of PSS every 20 min. To determine vascular contractility, the rings were incubated with high-potassium PSS containing (in mM): 74.7 NaCl, 60 KCl, 14.9 NaHCO_3_, 1.6 CaCl_2_, 1.18 KH_2_PO_4_, 1.17 MgSO_4_, 5.5 glucose, and 0.026 EDTA (pH 7.4). After adequate washout with PSS (x3), a concentration–response curve was made to the endothelium-dependent vasodilator acetylcholine (Ach, 10^−9^ to 10^−5^ M, accumulative) and a concentration-response curve to the endothelium-independent vasodilator nitroglycerin (NTG, 10^−9^ to 10^−5^ M) for each ring after submaximal precontraction with phenylephrine (PE, 10^−6^ M) as described [12].

### 2.8. Statistical Analysis

All data were presented as mean ± SEM, and analyzed using an unpaired, two-tailed Student’s *t*-test or two-way ANOVA followed by the Bonferroni correction. All statistical analyses were performed using Prism 8.0 software (GraphPad Software Inc., La Jolla, CA, USA). A two-sided *p* < 0.05 was considered statistically significant.

## 3. Results

### 3.1. Evaluation of Chronic DSS-Induced Colitis Models

**Figure 2:**
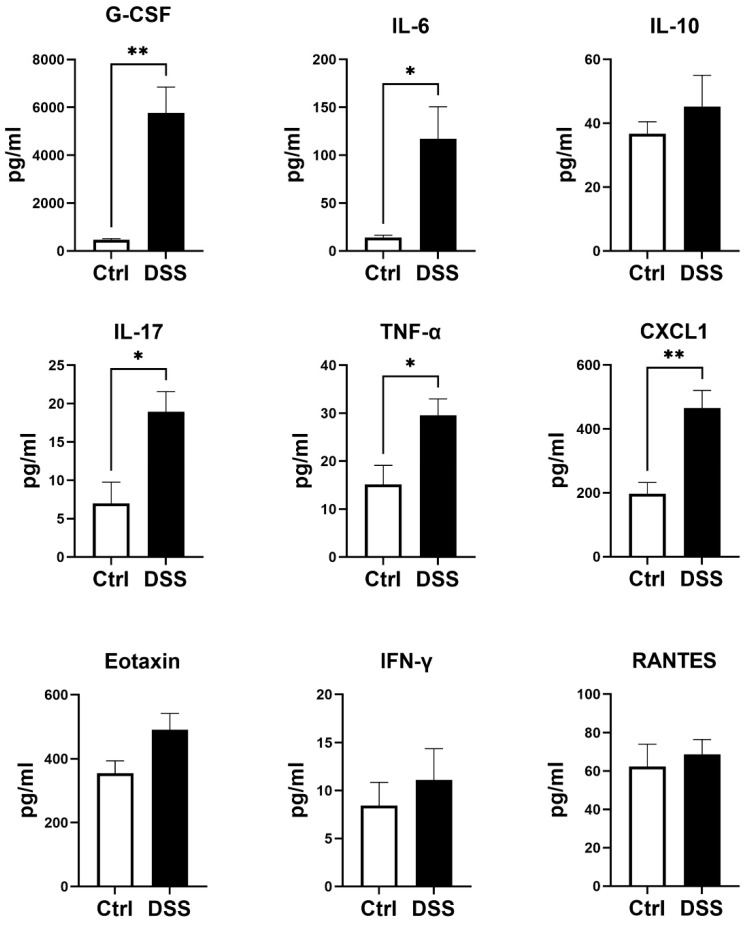
Levels of plasma cytokines in colitis model. Results are expressed as mean ± SEM. * *p* < 0.05, ** *p* < 0.01, by unpaired 2-tailed Student’s *t*-test, *n* = 5 mice in each group. G-CSF, granulocyte colony-stimulating factor; TNF-α, tumor necrosis factor alpha; IFN-γ, interferon gamma; IL, interleukin; CXCL1, chemokine ligand 1; RANTES, regulated upon activation, normal T cell expressed and presumably secreted.

Chronically DSS-treated mice developed significant chronic colitis, as demonstrated by fluctuating body weight (Figure 1B) together with a fluctuating DAI scoring (Figure 1C), and significant decreases in colon length (Figure 1D). Marked tissue damage and lamina propria inflammatory cell infiltration were evident in colon tissues of the colitis model, compared to the controls (Figure 1E). In addition, the plasma levels of granulocyte colony-stimulating factor (G-CSF), IL-6, IL-17, TNF-α, and chemokine ligand 1 (CXCL1) were significantly increased in the colitis model (Figure 2).

### 3.2. Blood Flow and Function Recoveries of the Ischemic Limb Were Preserved in Mice with Chronic Colitis

**Figure 3:**
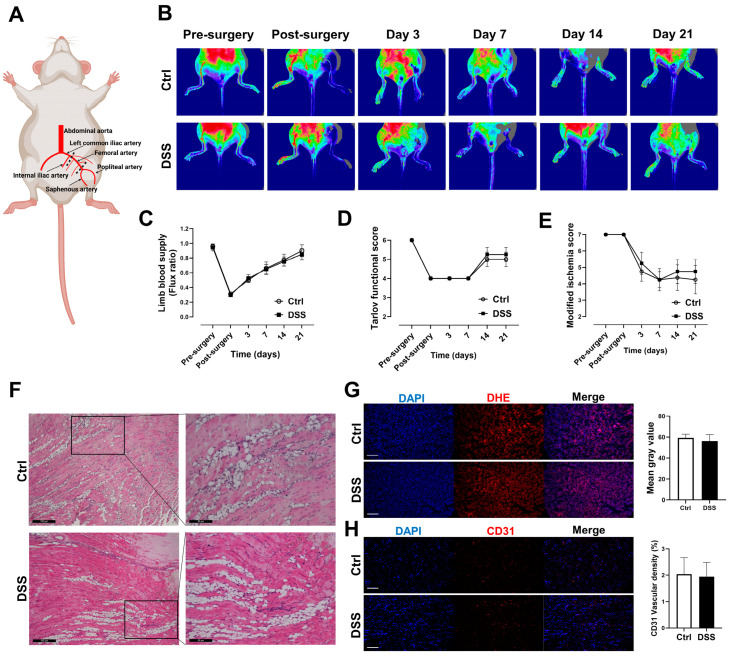
No significant changes in limb ischemia recovery, vascular density, and ROS levels in chronic colitis model. (**A**) Anatomy schematic of unilateral femoral artery ligation. (**B**,**C**) Analysis of laser doppler perfusion images for limb blood supply. (**D**,**E**) Functional score and ischemia score to assess limb function. (**F**) Representative images of H&E staining of gastrocnemius muscle from ischemic limb (scale bar, 100 μm and 50 μm). (**G**) Representative images of ROS in gastrocnemius muscle from ischemic limb using DHE staining (scale bar, 50 μm) and graph for quantification of ROS levels in muscle. (**H**) Representative images of vascular density in gastrocnemius muscle from ischemic limb using CD31 immunofluorescent antibody (scale bar, 50 μm) and graph for quantification of vascular density in muscle. Results are expressed as mean ± SEM. *n* = 7 mice in each group.

To determine if experimental chronic colitis could affect the recovery of ischemic limb, a unilateral hind limb ischemia model was produced by femoral artery ligation (Figure 3A), and laser doppler perfusion imaging of ischemic limb was conducted at baseline (before surgery at day 33) and day 33, 36, 40, 47, 54 after DSS administration (Figure 3B). There were no differences in blood flow recovery (ischemic limb blood perfusion/normal limb blood perfusion) (Figure 3C), functional score (Figure 3D), and ischemia score (Figure 3E) in mice with and without colitis. H&E staining showed marked fatty degeneration and atrophy in gastrocnemius muscle of the ischemic limb in mice with or without colitis (Figure 3F).

### 3.3. No Differences in Tissue ROS Level and Vascular Density in Mice with Chronic Colitis

Chronic colitis triggers a systemic inflammation with a potential increase in ROS production. Thus, frozen gastrocnemius muscle sections were prepared for ROS measurement using a DHE assay. To our surprise, there was no significant difference in ROS level in the freshly prepared ischemic gastrocnemius muscle cross-sections in mice with or without colitis (Figure 3G). To assess vascular density in the gastrocnemius muscle of the ischemic limb with or without colitis, gastrocnemius muscle frozen sections were incubated with anti-mouse CD31 immunofluorescent antibody. No difference in vascular density was observed in the gastrocnemius muscle of the ischemic limb in mice with or without colitis (Figure 3H).

### 3.4. Femoral Artery Endothelium-Dependent and -Independent Vasodilation Were Preserved in Mice with Chronic Colitis

**Figure 4:**
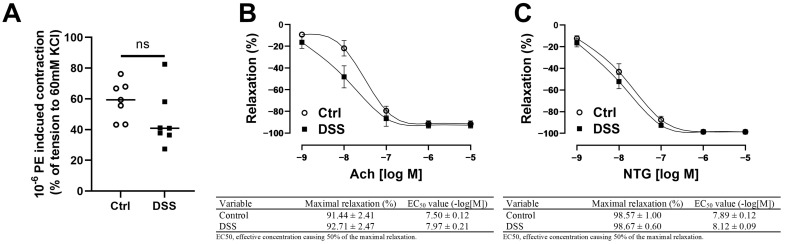
Femoral artery contractility and endothelial function were preserved in the chronic colitis model. (**A**) PE-induced contractility of femoral artery. (**B**) Ach-induced endothelium-dependent relaxation of femoral artery, maximal relaxation and EC50 for Ach were calculated in the table. (**C**) NTG-induced endothelium-independent relaxation of femoral artery, maximal relaxation and EC50 for NTG were calculated in the table. Results are expressed as mean ± SEM. By unpaired 2-tailed Student’s *t*-test (**A**), in two-way ANOVA followed by Bonferroni correction(**B**,**C**), *n* = 7 mice in each group. PE, phenylephrine; Ach, acetylcholine; NTG, nitroglycerin.

To determine if experimental colitis could negatively affect femoral artery endothelial function of the contralateral limb in mice, endothelium-intact rings of the femoral artery were assessed for endothelium-dependent and endothelium-independent relaxation after submaximal contraction with PE. There were no differences in PE-induced submaximal precontraction (Figure 4A), Ach-induced endothelium-dependent relaxation (Figure 4B), or NTG-induced endothelium-independent relaxation in mice with or without colitis (Figure 4C).

## 4. Discussion

In this study, we demonstrated that: (1) DSS treatment successfully induced chronic colitis in mice with significant increases in plasma proinflammatory cytokines; (2) no significant difference in blood flow recovery of ischemic limb in mice with and without chronic colitis; (3) no significant changes in ROS levels and vascular density in gastrocnemius muscle of the ischemic limb in mice with chronic colitis; and (4) no significant changes in endothelium-dependent and endothelium-independent relaxation of contralateral limb femoral artery in mice with chronic colitis.

CVDs remain the major cause of morbidity and mortality despite aggressive treatment of traditional risk factors [21]. Chronic inflammation plays a key role in the initiation and progression of CVDs. Indeed, a variety of systemic inflammatory diseases, such as systemic lupus erythematosus, rheumatoid arthritis, and psoriasis, are associated with an increased risk of CVDs and premature CVDs [22]. Similarly, patients with IBD also have an increased risk of CVDs over controls [3,23]. PAD, a form of CVD with reduced blood flow to peripheral arteries, is associated with atherosclerosis and vascular inflammation [24]. In a Chinese population-based cohort study, it was reported that the risk of PAD was increased in patients with IBD [25]. A French cohort study also showed that the risk of PAD was significantly increased in patients with IBD compared with the general population with the highest relative risk in those younger than 35 years [4].

Several inflammatory biomarkers, including C-reactive protein, TNF-α, IL-6, IL-17, and calprotectin, are significantly increased in patients with IBD [7]. Systemic inflammation, as indicated by TNF-α gene expression levels in peripheral blood monocytes, is associated with impairment in walking time in patients with PAD [26]. In addition, circulating Th17-associated cytokines (IL-6, IL-17, and TNF-α) are correlated to the severity and progression of carotid artery plaques [27]. The data from the present study also showed that the plasma levels of proinflammatory cytokines, G-CSF, IL-6, IL-17, TNF-α, and CXCL1, were significantly increased in mice with chronic colitis, suggesting the presence of significant systematic inflammation in mice with chronic colitis. However, there were no differences in tissue ROS levels in the ischemic limbs of mice with and without colitis.

Inflammation and angiogenesis have long been coupled together in IBD. Microvascular density assessed by CD31 staining was increased in IBD mucosa, and IBD mucosal extracts induced a potent angiogenic response in corneal angiogenesis assay [28]. TNF-α is a major inflammatory mediator that is involved in angiogenesis [29]. Studies have demonstrated TNF receptor-deficient mice exhibited an enhanced hind limb angiogenesis, while a reduced angiogenesis was observed in TNFR2-knockout mice [30]. Notably, it has been reported that sarcopenia is commonly present in the IBD population [31]. Increased serum levels of TNF-α are associated with muscle impairment by promoting protein degradation through the muscle-specific ubiquitin ligases [32]. The data from the present work demonstrated no difference in vascular density in the gastrocnemius muscle of the ischemic limbs in mice with or without colitis.

Endothelial dysfunction and arterial stiffness are considered among the important factors for the initiation and progression of CVDs. Chronic inflammation has been known to be associated with endothelial dysfunction [33]. Our recent meta-analysis demonstrated that patients with IBD were significantly associated with endothelial cell dysfunction, increased arterial stiffness, and carotid intima-media thickness [10]. In the present study, we observed that plasma TNF-α, IL-6, and IL-17 were significantly increased in mice with colitis. IL-6 and TNF-α are considered potent oxidative stress-inducing agents, thus leading to endothelial dysfunction [34,35]. IL-17 may induce endothelial dysfunction through activating RhoA/Rho-kinase [36]. Significant decreases in retinal blood flow have been reported in mice with T-cell transfer colitis and DSS-induced colitis [37,38]. Recent animal studies showed that the perivascular sensory neurotransmitter function of mesenteric arteries is significantly impaired in an IL-10 knockout mouse colitis model [39]. Recently, we demonstrated that abdominal aortic endothelial function was impaired selectively in female mice with chronic colitis [12]. To our surprise, the data from the present work showed that no significant changes in endothelium-dependent and endothelium-independent relaxation of the contralateral limb femoral artery were observed in the colitis model.

There were several limitations in the present study. First, only female mice were used based on the data from our recent study. Although abdominal aortic endothelial dysfunction occurs selectively in female mice, it is possible that endothelial dysfunction and/or impaired ischemic limb recovery may also occur in male mice with chronic colitis. Second, disease duration of the animal model could be an important factor for endothelial dysfunction of the femoral artery and ischemic limb recovery. Thus, a longer time point may be needed to demonstrate the effect of IBD on endothelial function of femoral artery and ischemic limb in the mouse model. Third, no studies were conducted to evaluate the potential protective effect of estrogen on limb ischemia recovery in the present study. Finally, there are several colitis models, such as the IL-10 knockout mouse model with spontaneous colitis, trinitrobenzene sulfonic acid-induced colitis, polyinosinic-polycytidylic acid-induced colitis, and T-cell transfer-induced colitis. These models could be used to determine if endothelial dysfunction and impaired recovery of ischemic limb could be demonstrated in IBD in the future.

In conclusion, despite demonstrating a successful implementation of the colitis model and the existence of a systemic inflammatory environment, DSS-induced chronic colitis had no significant impact on femoral artery endothelial cell function or the recovery of ischemic limbs in female mice.

## Data Availability

Not applicable.
